# A Complete Data Set of Equatorial Projections of Saturn's Energetic Neutral Atom Emissions Observed by Cassini‐INCA

**DOI:** 10.1029/2020JA028908

**Published:** 2021-06-03

**Authors:** A. Bader, J. Kinrade, S. V. Badman, C. Paranicas, D. A. Constable, D. G. Mitchell

**Affiliations:** ^1^ Department of Physics Lancaster University Lancaster UK; ^2^ Johns Hopkins University Applied Physics Laboratory Laurel MD USA

## Abstract

Observations of energetic neutral atoms (ENAs) are a useful tool for analyzing ion and neutral abundances in planetary magnetospheres. They are created when hot plasma, originating for example from magnetic reconnection sites, charge‐exchanges with the ambient neutral population surrounding the planet. The motion of ENAs is not governed by the magnetic field, allowing remote imaging. During the Cassini mission, the Ion and Neutral CAmera (INCA) of the Magnetosphere Imaging Instrument (MIMI) collected vast amounts of hydrogen and oxygen ENA observations of Saturn's magnetosphere from a variety of different viewing geometries. To enable investigations of the morphology and dynamics of Saturn's ring current, it is useful to re‐bin and re‐project the camera‐like views from the spacecraft‐based perspective into a common reference frame. We developed an algorithm projecting INCA's ENA observations into a regular grid in Saturn's equatorial plane. With most neutrals and ions being confined into an equatorial rotating disc, this projection is quite accurate in both spatial location and preservation of ENA intensity, provided the spacecraft is located at large enough elevations. Such projections were performed for all INCA ENA data from the Cassini Saturn tour; the data are available for download together with a Python routine flagging contaminated data and returning detailed spacecraft geometry information. The resulting data set is a good foundation for investigating for example the statistical properties of Saturn's ring current and its complicated dynamics in relation to other remote and in situ observations of, for example, auroral emissions and magnetotail reconnection events.

## Key Points

- All Ion and Neutral CAmera observations of energetic neutral atoms from the Cassini Saturn tour are projected into Saturn's equatorial plane
- The full set of projections in the form of daily .fits files is accessible online for further scientific analyses
- Different types of data contamination are identified and projections are flagged accordingly when loaded with the Python routine provided

## 1 Introduction

Energetic neutral atom (ENA) imaging is a useful technique for analyzing the interaction between hot space plasmas and neutral gas in planetary magnetospheres. Energetic ions can undergo charge exchange with a neutral population ‐ the planetary exosphere, for example ‐ to create an ENA particle that carries information on the original ion's energy and direction of motion. This ENA continues on its trajectory, unaffected by electromagnetic fields, and can be observed by a remote detector. Remote sensing of an ENA population hence provides spectral, compositional, and pitch angle information of the source plasma. As the observed ENA flux is a line of sight integral dependent on both neutral and ion densities and their charge‐exchange cross‐section, dynamics and gradients in ENA intensity may reflect not only dynamics of the ion source population but also the underlying neutral gas population (see, e.g., Brandt et al., 2018, and references therein).

While there were several instruments observing the terrestrial system, the Cassini orbiter carried the so far only ENA imager reaching the outer planets of our solar system. A flyby at Jupiter revealed ENA emissions from both the planet's upper atmosphere and from a torus just outside the orbit of the icy moon Europa (e.g., Mauk et al., 2003; Mitchell et al., 2004), details of which will be revealed by observations from the upcoming JUpiter ICy moons Explorer (JUICE; Grasset et al., 2013). Just recently, Mauk et al., 2020 demonstrated that the Juno spacecraft's Jupiter Energetic particle Detector Instrument is also capable of observing reasonable ENA foreground if the population of energetic charged particles entering the detectors does not obscure the signal (e.g., as Juno moves through high southern polar latitudes). This allowed for distinct observations of ENA emission from the Io orbit, Europa orbit, and Jupiter itself.

Upon reaching the Saturn system, years of ENA observations revealed a highly dynamic magnetosphere driven both internally through Saturn's rapid rotation and externally through interaction with the solar wind. First observations revealed injections of energetic plasma from the nightside, thought to be related to magnetotail reconnection events and similar to substorms in the terrestrial magnetosphere (Mitchell et al., 2005). The injected plasma is typically observed to corotate with Saturn for up to a full planetary rotation or longer (e.g., Carbary & Mitchell, 2014; Mitchell, Krimigis, et al., 2009), continuing to produce ENA emissions and forming the ring current.

Several studies have discovered periodicities in the ENA intensity indicative of rotational modulation near the planetary rotation period (Carbary et al., 2008, 2011; Carbary & Mitchell, 2017; Krimigis et al., 2005; Paranicas et al., 2005). These may be caused by the periodic thinning and thickening of Saturn's equatorial plasma sheet by planetary period oscillation systems, two rotating systems for field‐aligned currents which originate near Saturn's poles and span its entire magnetosphere (e.g., Cowley & Provan, 2017; Jackman et al., 2016; Provan et al., 2018). This thickness modulation is also observed in the statistical ENA intensity (Kinrade, Bader, et al., 2020) and may trigger periodic reconnection in the magnetotail (e.g., Bader et al., 2019; Bradley et al., 2018, 2020).

ENA emissions can often be mapped directly to ultraviolet auroral emissions through field‐aligned current systems connecting Saturn's polar auroral ionospheres to the equatorial ring current plasma (Kinrade, Badman, et al., 2020; Mitchell, Krimigis, et al., 2009). While observations of ion and electron beams on auroral field lines have been studied occasionally (Bader et al., 2020; Mitchell, Kurth, et al., 2009), holistic investigations of the relation between ENAs and the aurorae are needed to gain a better understanding of the physical process linking the two.

While many studies analyzed Cassini INCA ENA observations to reveal the intriguing and complex dynamics of Saturn's magnetosphere, much of the INCA data obtained during Cassini's 13 years in orbit around Saturn is left nearly unexplored. This is partly due to the highly variable viewing geometry and data quality of the ENA observations, complicating especially the implementation of statistical studies. Our work is intended to remove some of these barriers by projecting all INCA data into Saturn's equatorial plane, which is where the majority of ions and neutrals are concentrated and where most ENAs are produced. This normalizes the data set to a common reference frame and takes care of proper data calibration and cleaning, making it easy to use and compare to other data sets.

In the next section, a broad description of the INCA instrument is given, followed by a detailed breakdown of the data calibration and projection procedures performed on its observations. Section 4 provides an overview of the data coverage throughout the Cassini mission, while Section 5 informs about the different types of contamination expected in the data set. In Section 6, we investigate the response of the INCA instrument to typical ENA fluxes, followed by a consideration of viewing geometry effects on the observed ENA intensity in Section 7. Finally, we summarize our findings in Section 8.

## 2 The MIMI‐INCA Instrument

The MIMI‐INCA detector (Krimigis et al., 2004) was a time of flight (ToF) sensor capable of measuring both ions and ENAs with energies from 7 keV to 3 MeV per nucleon, providing information on both the mass and the direction of motion and energy of each detected particle. INCA had an instantaneous field‐of‐view (FOV) of 120° × 90° and an angular resolution of up to 64 × 64 pixels, that is, (120/64)° × (90/64)°. When operated in ion mode, INCA was hence able to resolve a significant part of the local ion pitch angle distribution, revealing for example field‐aligned ion beams and conics on auroral field lines (Bader et al., 2020; Mitchell, Kurth, et al., 2009).

Collimator plates mounted at the detector entrance could be charged to a large potential, deflecting incoming charged particles and preventing them from entering the detector. With this potential applied, only ENAs could be observed—providing remote imagery of the global distribution of energetic ions using a technique known as ENA imaging (e.g., Roelof, 1987).

Incoming particles triggered the ToF sensor by penetrating a thin start foil, producing secondary electrons which entered the start multichannel plate (MCP) to provide the start time. The original incident particle then traveled further through the instrument toward the end foil; upon impact secondary electrons were produced and recorded via the stop MCP. The particle's velocity could be obtained using its travel time through the instrument and the distance between the start and stop foils. The mass of an incident particle was determined by the pulse height of the MCP signal, as the number of secondary electrons produced increases significantly with the particle mass. The pulse height was sufficient to distinguish between the two most common neutrals in Saturn's magnetosphere, hydrogen and oxygen, but further mass discrimination was not feasible. The particle energy could be obtained using the mass and velocity determined from the measurements, and the direction of motion could be inferred from the particle's impact location on the MCP.

To produce sensible observations even when the spacecraft's attitude was not stable, INCA was equipped with two internal motion compensation algorithms. These registered counts observed over the time of an exposure in an inertial coordinate image buffer using quaternions transmitted to INCA from the spacecraft attitude system to take into account any spacecraft motions.

When the spacecraft was rolling about its *Z*‐axis (which it did for several hours during many of the data downlink intervals), the flight software (FSW) knew where INCA's FOV was moving and so could keep a continuous de‐spun buffer of “sky” pixels into which counts were accumulated. Every 360° spin was divided into four quadrants, each of which was transferred in telemetry frames to the spacecraft for downlink after INCA had fully populated it. Since for a given sky pixel the amount of time spent exposed to each instrument pixel was well known, the application of the flat‐fielding matrix was well determined such that all sky pixels could be accurately weighted by the instrument response to obtain ENA intensities.

In the other instance the FSW did not know where the spacecraft would be moving; so while counts could still be accumulated in inertial “sky” pixels, the weighting applied to them for conversion to intensity was only approximated. In general, a sky pixel area larger than the INCA FOV would have counts accumulated in it, and the FSW would choose the centroid of that distribution that presumably contained the highest exposure times and transmit this data into a telemetry frame. The time spent on each pixel was also retrievable from the quaternions. At the end of the exposure period, an image corresponding to the size of the INCA FOV was extracted, centered on the average INCA boresight position. This approach was useful when the spacecraft was either not articulating (in which case the instrument coordinate system and the inertial sky system were identical), or when the optical remote sensing instruments were producing mosaic images of a moon, Saturn, or the rings, which typically meant rastering with rotations back and forth about the spacecraft *Z*‐axis interspersed with steps in angle about the spacecraft *X*‐axis. Typically these motions were on the order of or less than a single INCA pixel in extent, and so the corrections between instrument and inertial systems were minimal. In cases where they were large (e.g., during slews between different objects), the INCA images were not generally very useful.

## 3 Data Calibration and Projection

The basis of our newly created data set is the uncalibrated MIMI‐INCA Planetary Data System (PDS) archive CO‐E/J/S/SW‐MIMI‐2‐INCA‐UNCALIB‐V1.1. We first select all data of interest by filtering for

- High‐resolution type: Spatial
- High voltage (HV) state: 1 (deflection voltage turned on, includes ENA and excludes ion observations)
- Particle species: H for hydrogen‐dominated, CNO for oxygen‐dominated images
- ToF channel: 7 for high ToF (low energy range), 0 for low ToF (high energy range)

This selection returns four types of ENA imagery:

- Hydrogen, ToF channel 7 (24–55 keV), 32 × 32 pixels
- Hydrogen, ToF channel 0 (50–90 keV), 64 × 64 pixels
- Oxygen, ToF channel 7 (90–170 keV), 32 × 32 pixels
- Oxygen, ToF channel 0 (170–230 keV), 64 × 64 pixels

In theory, all four image types are obtained during the same time, resulting in matching start and stop times for each exposure. However, we found that the HV state flags did sometimes not agree at the beginning or end of ENA imaging sequences, such that, for example, hydrogen images were registered with HV state 1 (ENA images) while oxygen images exposed at the same time were registered with HV state 0 (ion images). These ambiguous cases were dropped from the data set before any further processing was performed.

The raw counts of each image are calibrated and converted to differential energy flux using

(1) $$\text{differential energy flux}=\frac{\text{counts}\times \text{flux factor matrix}\times \text{saturation ratio}}{\text{exposure time}\times \text{energy width}},$$

where

- differential energy flux is in units of particles/cm^2^/s/sr/keV.
- counts is the raw number of counts from the PDS archive.
- flux factor matrix is the inverse of the pixel‐wise product of the instrument geometric factor and efficiency, a matrix of the same size as the detector grid in units of/cm^2^/sr—different for each ENA image type and depending on observation mode (spinning vs. staring) as well as time of observation (efficiency degraded throughout the mission). These matrices are contained in the calibration files accompanying the raw data in the PDS archive.
- saturation ratio is the number of valid ToF events divided by the number of events processed by the processing unit. This ratio is applied to reduce saturation effects.
- exposure time is the accumulation time of the image in seconds.
- energy width is the energy width of the ToF channel in keV.

These images are in the frame of the INCA detector; their perspective is hence dependent on spacecraft position and attitude. To remove this dependency, we project all observations into Saturn's equatorial plane to obtain a consistent set of easily comparable observations. This projection is performed separately for each INCA exposure to minimize inaccuracies arising from spacecraft motion and attitude changes.

We first determine Cassini's position and the INCA pointing at the central time of each exposure using the NAIF SPICE toolkit. Care needs to be taken to account for INCA's internal motion compensation, which may add offsets of an integer number of pixels in both the long and short dimensions of the INCA FOV. Once the INCA pointing has been adjusted accordingly, the geometric projection is done by tracing the line of sight of each INCA pixel into Saturn's equatorial plane. To conserve the shape of each INCA pixel in the projection, we trace its four corner view vectors as well as several support vectors along each pixel edge. The intersection points of all these view vectors in the equatorial plane are then connected to form a polygon whose intersection with the base projection grid is determined.

**Figure 1:**
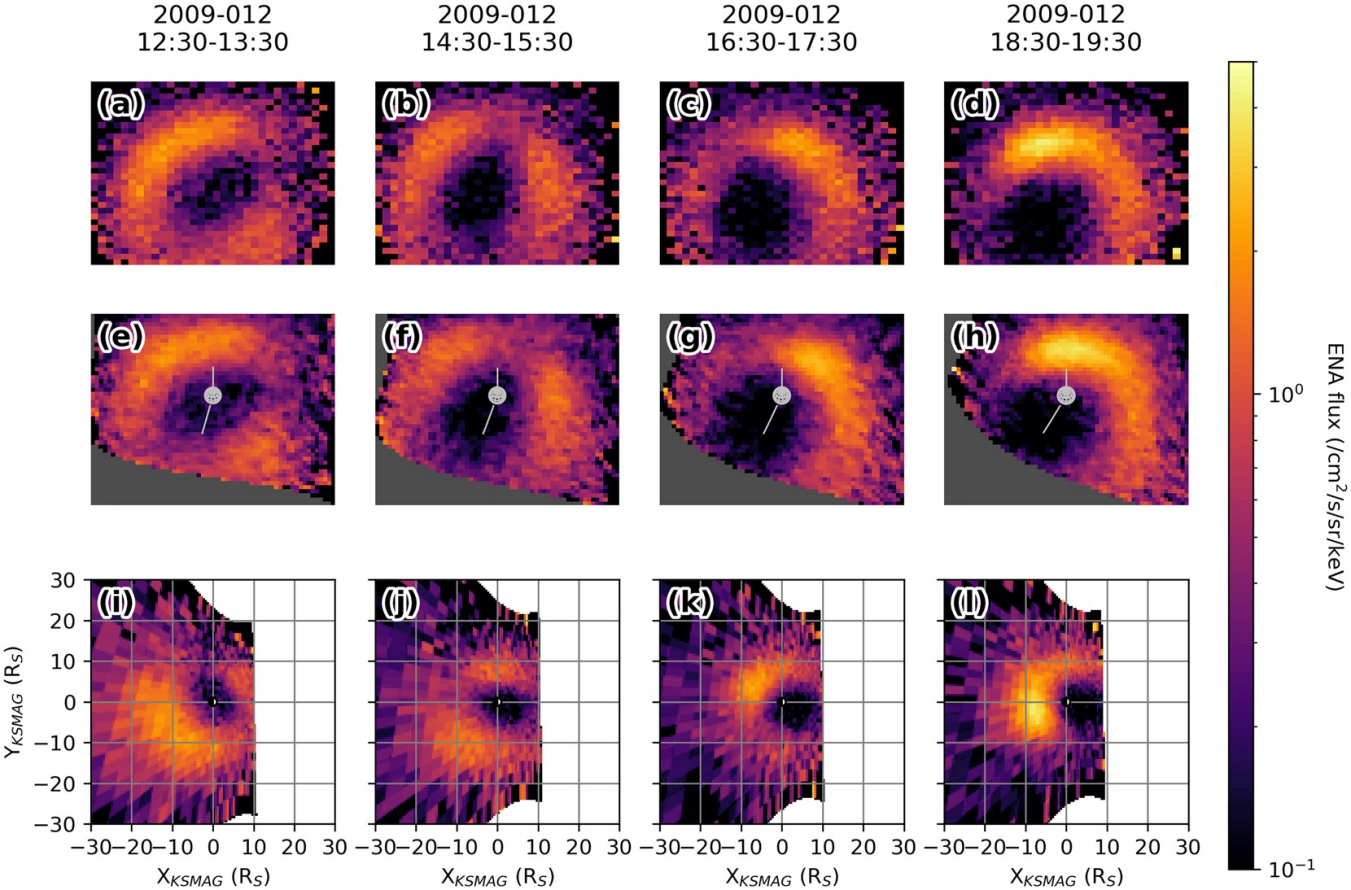
Series of Cassini MIMI‐INCA observations of hydrogen ENAs between 24 and 55 keV, averaged over 1 h of exposure time. (a–d) ENA fluxes in the original detector grid. (e–h) Re‐centered onto Saturn and rotated such that the north pole is up. The vertical line pointing upwards from the planet model indicates Saturn's rotation axis (Z_*KSMAG*_), the other line indicates the X_*KSMAG*_ axis directed toward the Sun. (i–l) Projections of these observations into Saturn's equatorial plane as seen from above the north pole, with the Sun to the right. During the observation window shown here, Cassini moved from (*X*, *Y*, *Z*) (7.7, 1.7, 12.7) *R*_*S*_ to (5.1, 2.9, 12.6) *R*_*S*_, essentially traversing high above the dayside magnetodisc toward Saturn's dusk side. ENA, energetic neutral atom; INCA, ion and neutral camera; MIMI, Magnetosphere Imaging Instrument.

**Figure 2:**
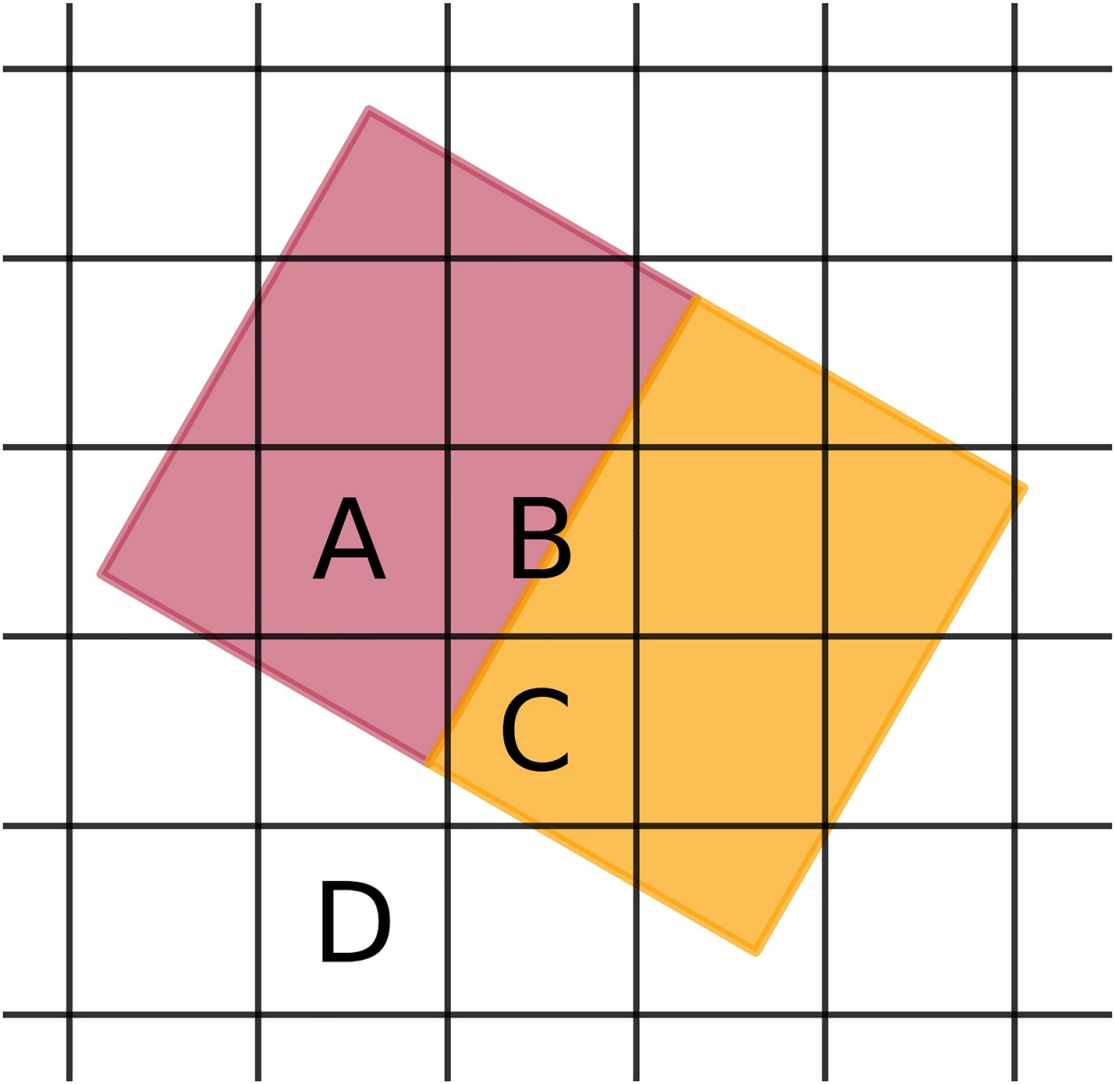
Sketch of two projected INCA pixels (colored boxes) intersecting the regular projection grid in the equatorial plane (black lines). Projection pixel A is located entirely within the purple projected INCA pixel polygon; it will simply be assigned the flux recorded by the purple INCA pixel. Projection pixel B on the other hand is intersected by the projections of both the purple and the orange INCA pixel; it will instead be assigned a flux value calculated from the purple and orange INCA pixels' recorded fluxes (mean value, weighted by the intersection area between the purple/orange INCA pixel and the projection pixel). The same applies for projection pixel C, where the final flux value is weighted by the relative size of the INCA pixels' intersection area and the white section (not intersected by an INCA pixel) is disregarded in the weighting. Finally, projection pixel D will not be assigned a flux value as it is not intersected by any INCA pixel. INCA, ion and neutral camera.

Projection grid pixels entirely within the projected INCA pixel polygon are assigned the flux value of this INCA pixel, while projection grid pixels (partly) intersected by several INCA pixels are assigned the intersection area‐weighted average of the intersecting INCA pixels' flux values; see Figure 2 for a graphical explanation. The projection grid is limited to ±30 *R*_*S*_ distance from the center of Saturn in both the X_*KSMAG*_ and Y_*KSMAG*_ axes. The resulting projections are then saved for each exposure separately, combined into one .fits file per day and per ENA observation type, together with some metadata detailing the observation time, Cassini's location, and some other parameters. These files are available for further analyses at https://doi.org/10.17635/lancaster/researchdata/384. An example of raw and projected observations can be seen in Figure 1, where the top row shows some observations in the original INCA pixel grid and the bottom row shows the final equatorial projections obtained from them.

## 4 Available Data

The INCA instrument performed ENA observations throughout most of Cassini's Saturn tour, from the first approach to the final plunge into the upper atmosphere, without major interruptions. However, the accuracy and validity of the projections described in this study are dependent on the observation geometry of an exposure.

The two parameters of importance here are the spacecraft's distance from and elevation above a projection pixel. The projected size of an INCA pixel increases with the square of the spacecraft distance and further depends on the cosine of the spacecraft elevation angle; lower elevation angles leading to increased stretching of pixels projected into the equatorial plane. Beyond certain spacecraft distances and/or elevation angles, the spatial resolution may hence be considered too low for reliable scientific analyses. On the other hand, data obtained too close to the equatorial plane may not produce representative ENA intensity values due to the detector being located within the ENA source region. We hence recommend careful consideration of the viewing geometry of each exposure.

It is to note that many observations may have “good” and “bad” sections; for example, with Cassini located at a reasonable distance above the dawn magnetodisc one could expect good resolution of the dawn, but stretched pixels with bad spatial resolution of the dusk ENA emissions. This means that instead of limiting the range of spacecraft distances and/or elevations relative to Saturn itself within which data are considered usable, it is preferable to limit the range of spacecraft distances and/or elevations relative to single projection pixels. The Python data loading function provided together with the equatorial projections is able to return the necessary geometric information on a per‐pixel basis.

**Figure 3:**
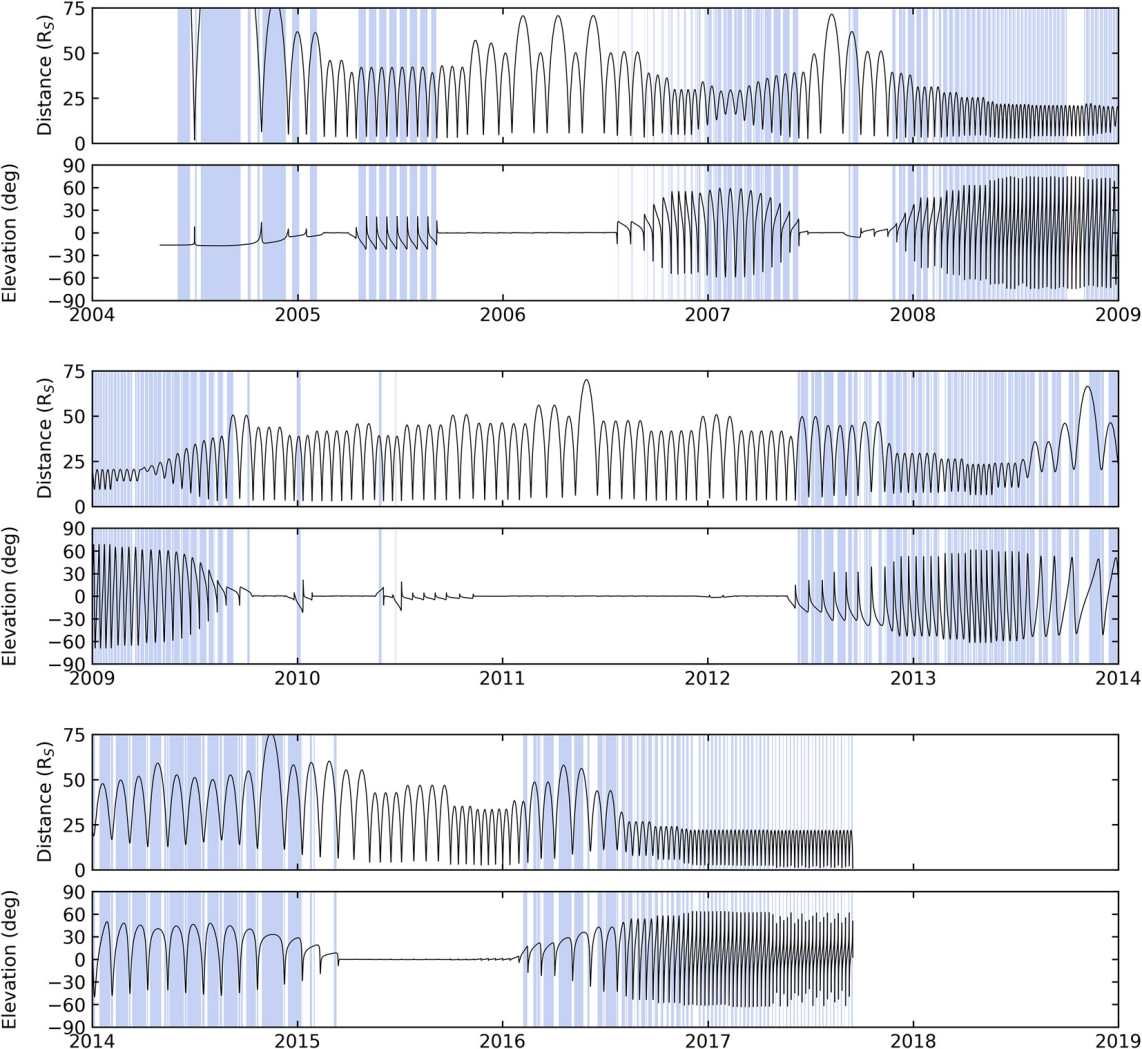
Timeline of Cassini's radial distance from Saturn and latitude above the equatorial plane through the entire Saturn tour. Blue shaded regions indicate days where MIMI‐INCA ENA observations were obtained while Cassini was located between 4 and 100 Saturn radii from the equatorial plane. ENA, energetic neutral atom; INCA, ion and neutral camera; MIMI, Magnetosphere Imaging Instrument.

To give a rough idea of the availability of useful equatorial ENA projections, Figure 3 shows Cassini's orbital distance and elevation (latitude) relative to Saturn throughout its entire Saturn tour. All days during which ENA observations were obtained while Cassini was located at distances between 4 and 100 Saturn radii from the equatorial plane are shaded blue. While we notice lengthy gaps without any valid equatorial projections during Cassini's equatorial orbits in 2006, 2010–2012, and 2015, there are plenty of observations available from higher latitude orbits spread throughout the remaining mission. The data set made available online includes projections of all mission data, regardless of viewing geometry.

## 5 Contamination Handling

Virtually no data set is free of bad data, and INCA data is no exception to that. Different kinds of contamination are known to occur in the INCA detector and should be removed from the data set before performing scientific studies. A Python routine for loading the projected INCA data is provided with the data set; it checks for all known kinds of contamination and flags possible events. We note that the contamination flagging is rather sensitive to make sure all contamination events are covered, so some data flagged as compromised may be usable—whether this is the case for a certain observation is left to the user to decide.

The first type of bad data is “out of calibration” events. These occur when the INCA start and stop voltages are not in the proper calibration range, which is often related to maintenance procedures but can also occur unplanned. A list of these events is provided by the instrument team together with INCA calibration files.

Data downlinked to Earth from the Cassini spacecraft can sometimes contain bit errors. These are usually identifiable as clear outliers in ENA intensity and are hence quite straightforward to filter out. Sunlight contamination similarly leads to enhanced intensities in affected detector regions and can be identified in the same way as bit errors; however, most or all of these events have already been removed from the data set by the instrument team.

Another type of contamination is the occurrence of ion beam signatures in ENA data. While a strong potential could be applied to INCA's collimator plates to prevent charged particles from entering the detector during ENA imaging phases, ions with energies >500 keV were still able to pass by design (Krimigis et al., 2004). However, even in the beginning of the mission ion beams could sometimes be observed to create signatures in ENA imagery when Cassini crossed auroral field lines. This indicated that either higher energy ions were present or the shielding was less efficient than assumed, or both. Moreover, a short developed in the high voltage circuit in 2015 such that the deflection potential could only reach half its intended strength, further reducing the performance of the ion deflector system. This means that especially toward the end of the Cassini mission many ENA observations contain features caused by ion beams.

**Figure 4:**
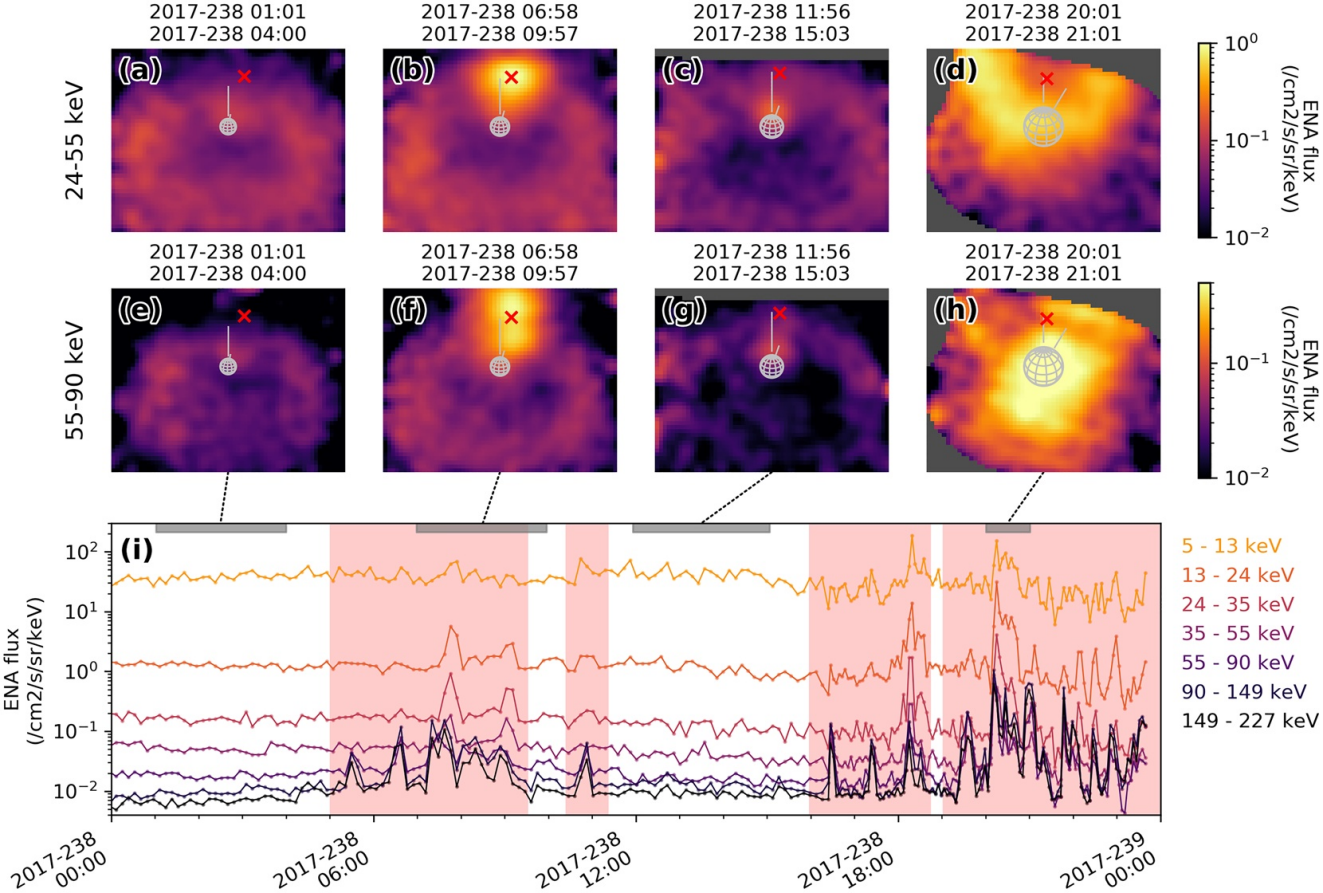
Example of an ion contamination event. (a–h) MIMI‐INCA hydrogen neutral mode observations in two different energy ranges, some of which exhibit clear intensifications in pixels close to the local magnetic field direction (red cross), in the last image even a well‐resolved ion conic. (i) High energy resolution time series, the exposure times of panels (a–h) highlighted with gray shading near the top. The contamination events are characterized by increased fluxes in the higher energy bins (darker colors), times shaded red were identified as contaminated by our detection algorithm. INCA, ion and neutral camera; MIMI, Magnetosphere Imaging Instrument.

An example of such contamination is shown in Figure 4. Shown are both INCA ENA images with high spatial resolution (Figures 4a–4h) and time series of high ToF resolution INCA observations (Figure 4i). Ion contamination events are visible as strong intensifications near the magnetic field‐aligned direction (red cross). In Figures  4b and 4f, we can clearly identify a field‐aligned ion beam whereas Figures 4d and 4h appear to show an ion conic. The high ToF resolution data in Figure 4i shows the measured “ENA” intensity in different energy ranges, and it is clear that ion signatures in ENA imagery correspond to sharp flux increases by sometimes more than an order of magnitude especially in the higher energy bins. The origin of these ion signatures is discussed in, for example, Mitchell, Kurth, et al. (2009), Badman et al. (2012), and Bader et al. (2020).

This property is used to identify ion contamination events throughout the entire Cassini mission. This is done by comparing the 10‐min mean intensity with the 12‐h median intensity recorded in the high ToF channels; all times where the 10‐min mean is larger than two times the 12‐h median in one of the 90–149 or 149–227 keV energy channels and the relative increase in this channel is larger than in the 5–13 keV channel are flagged as likely ion contamination events.

The last type of bad data is the frequent occurrence of enhanced fluxes at the start and end of an ENA imaging sequence. These enhancements are likely caused by observations being classified as “HV state 1” even though the deflection voltage was in the process of ramping up or down after or before ion observations were performed, leading to ions entering the detector and increasing the recorded fluxes. These events are identified in a similar fashion as ion contamination events described in the paragraphs above, bar the constraint of higher energy channels needing to show a larger flux increase than the lowest energy channel.

## 6 Instrument Response and Effects of the Projection on the Flux Histogram

Before performing scientific analyses on these data it is important to understand the caveats of INCA observations and the effects of performing projections as described in this study. Typical INCA exposures take some minutes to obtain, and during that time a single pixel usually collects at most a handful of hydrogen 24–55 keV counts. At higher energies, the number of counts detected is certainly even lower, such that special care has to be taken when producing statistical averages such as shown in Kinrade, Bader, et al. (2020).

**Figure 5:**
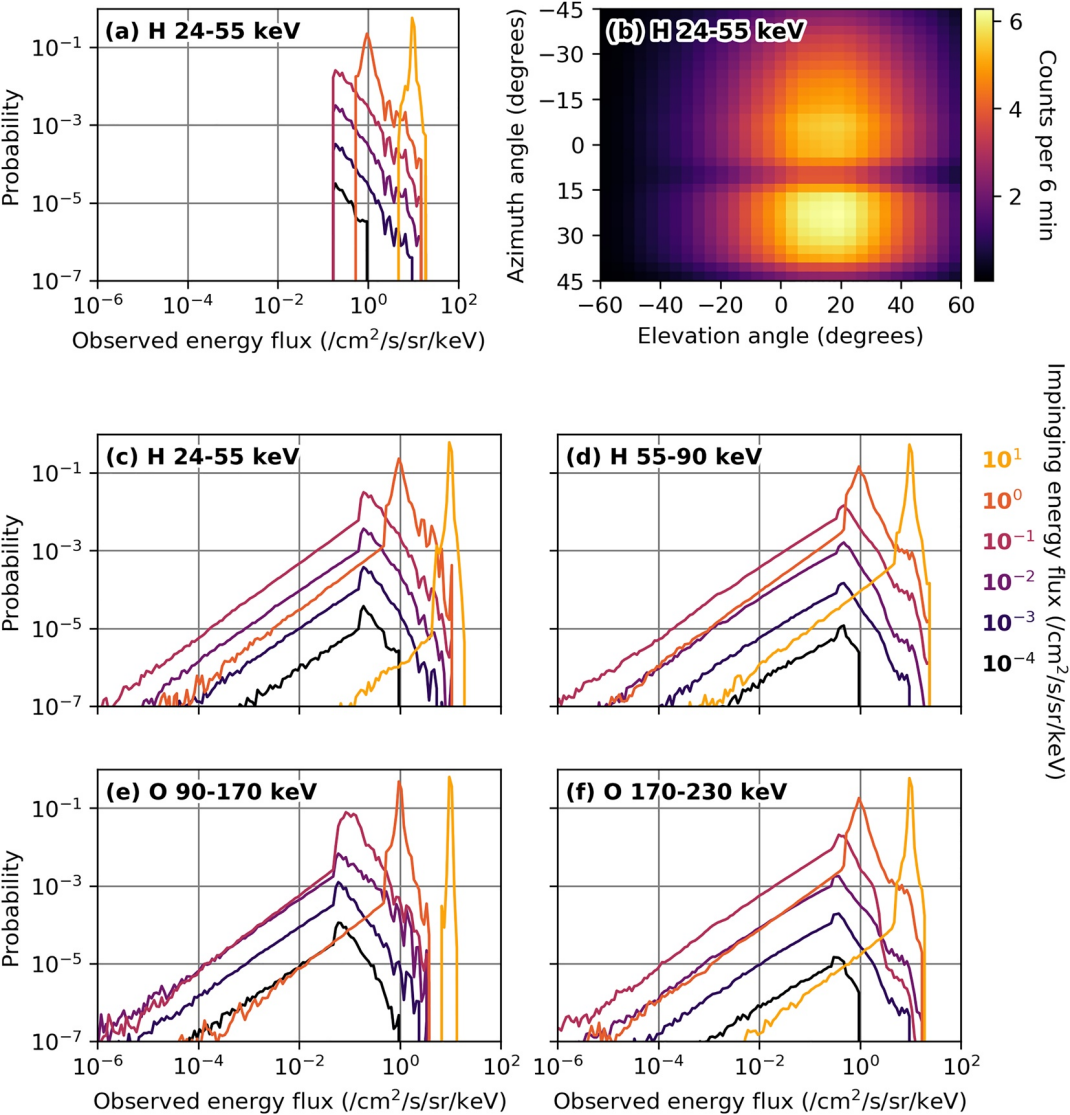
(a) Modeled INCA histograms for different constant incident fluxes. (b) INCA pixel sensitivity for a flux of 1 particle/cm^2^/s/sr/keV. (c–f) Histograms after rebinning of modeled INCA data, for hydrogen and oxygen in their respective energy ranges. INCA, ion and neutral camera.

To illustrate this, we reverse‐engineered the number of counts expected within a typical 6 min exposure from the instrument calibration procedure for a given ENA flux (see Figure 5b, showing the expected counts for a flux of 1 hydrogen particle/cm^2^/s/sr/keV in the 24–55 keV range). From these theoretical (non‐integer) counts, a set of 10,000 observations with integer counts was created—that is, for a pixel that should observe 0.76 counts in one 6‐min window, 7,600 observations with one count, and 2,400 observations with 0 counts. Calibrating these theoretical observations with the typical routine results in certain histogram shapes which are shown in Figure 5a.

We notice that in this case, for H 24–55 keV, high fluxes of 1 or 10 particles/cm^2^/s/sr/keV still result in a clear histogram peak at these ENA flux values, respectively. However, all lower fluxes have the same histogram shape which is entirely determined by the relative sensitivities of the detector pixels; the lowest measurable flux corresponds to one count in the most sensitive INCA pixel and is noticeable as a clear vertical boundary below which all histogram bins are empty (apart from the zero flux bin which is not shown here). At these fluxes, the real flux value is still determined to high accuracy even though there are no real histogram counts associated with it; the average strongly depends on the ratio of nonzero to zero detector count events. Statistical measures like the median or percentiles are hence meaningless and only the mathematical mean is a useful statistic.

The real data histograms, however, show observations of much lower fluxes, many orders of magnitude below the lowest fluxes observable by the detector (see also Kinrade, Bader, et al. [2020]). This is an effect of the projection procedure—one pixel in the projection grid may be intersected by more than one INCA pixel. The flux value assigned to this projection pixel then depends on the flux values of the intersecting INCA pixels and the relative area which is intersected. A zero‐flux INCA pixel and a neighboring nonzero‐flux INCA pixel intersecting the same projection pixel hence average to a flux value somewhere in between these two, depending on the ratio of intersected areas, which is eventually assigned to the intersected projection pixel. The effect of this was modeled by rebinning the theoretical data whose histogram is shown in Figure 5a onto a smaller, randomly shifted grid. We observed that this creates an exponential tail toward low fluxes in the histograms, visible as a straight line in the log‐log scaled histograms in Figures 5c–5f and in good agreement with the real data shown in Kinrade, Bader, et al. (2020).

## 7 Observation Geometry Effects: Distance Scaling and Compton‐Getting Effect

Finally, we investigate how ENA intensities recorded by INCA are influenced by the viewing geometry at the time of the observations. We hereby focus on two parameters: the line of sight distance between the instrument and the observed pixel in the equatorial plane, and the angle between the corotational plasma flow in the source region and INCA's viewing direction.

**Figure 6:**
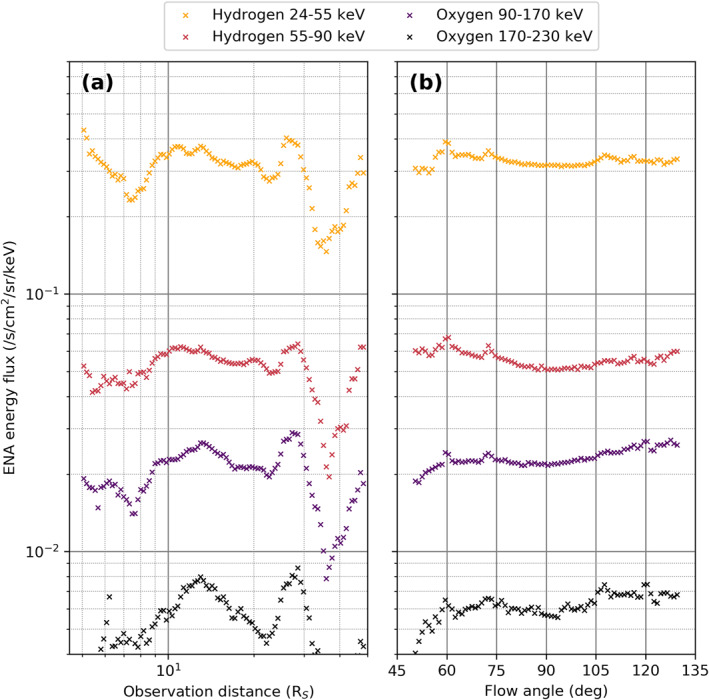
Mean ENA intensity observed between 7 and 11 *R*_*S*_ distance from Saturn's center in the equatorial plane and at spacecraft elevation angles >50°. (a) Binned by spacecraft line of sight distance. (b) At spacecraft line of sight distances between 5 and 30 *R*_*S*_, observed at different “flow angles”. Flow angle is defined as the angle between the corotational particle flow in the ENA source region in the equatorial plane and the viewing direction of INCA. 0° flow angle hence corresponds to flow pointed away from INCA viewing direction and 180° to flow directly into the INCA detector. ENA, energetic neutral atom; INCA, ion and neutral camera.

This is done by selecting contamination‐free observations of the ring current at radial distances between 7 and 11 *R*_*S*_ obtained from spacecraft elevations >50° (above the observed pixel itself, not above the center of Saturn). These are then binned either by line of sight distance of the spacecraft from the source region or by “flow angle”, the angle between the corotational particle flow in the ENA source region in the equatorial plane and the viewing direction of INCA. Figure 6 shows the average ENA intensity depending on both of these parameters.

We notice that the spacecraft distance does not strongly affect the average ENA intensity (see Figure 6a). This may be surprising; one would rightly expect the ENA intensity to decrease ∼*r* ^−2^. However, while the physical intensity of a given part of the source region decreases at this rate, the size of the source region observed within one INCA pixel increases with ∼*r* ^2^ such that these two changes approximately cancel. This is only true for as long as a single INCA pixel captures a sufficiently homogenous ENA source region, but breaks down at larger distances—eventually INCA cannot properly resolve the ring current and an increase in pixel FOV will not increase the size of the ENA source observed within this pixel anymore; the ENA emitter is then more akin to a point source and the observed intensity should decrease roughly as ∼*r* ^−2^ as was shown in, for example, Paranicas et al. (2005) where the total image average intensity was investigated.

Much of the variability visible in Figure 6a is certainly attributable to temporal changes in the ENA source intensity and orbital bias. As most of the selected observations from Cassini's Saturn tour were taken between roughly 10 and 20 *R*_*S*_ instrument line of sight distance, the average values shown are relatively smooth in between these distances but quite variable outside where far less observations are available and the average is likely to be more biased toward certain observation geometry or magnetospheric conditions.

Focusing on spacecraft distances between 10 and 20 *R*_*S*_, we find that the mean hydrogen 24–55 keV intensity shows only a slight decrease with increasing spacecraft distance, which appears to steepen for higher energies and heavier particles. Only for oxygen in the 170–230 keV energy band does this decrease become significant though, likely a product of INCA's decreased spatial resolution in this observation mode combined with the sparsity of such high energy particle counts which leads to observation characteristics more akin to spatially and temporally separate point sources rather than actual distributions of particles observed at lower energies.

Figure 6b shows how the mean ENA intensity depends on the angle between the source plasma motion relative to the detector viewing direction. It is important to investigate this dependence to roughly quantify the overall impact of the Compton‐Getting (CG) effect on the INCA data set. The CG effect is a consequence of *E* × *B* and gradient‐curvature drifts influencing the motion of energetic charged particles as they gyrate around magnetic field lines (Gleeson & Axford, 1968). A small “toward” or “away” motion of the plasma relative to the spacecraft shifts the detected energy of an ENA only slightly but in a power‐law type spectrum this is enough to cause a systematic measurement offset, known as the CG effect. Specifically for INCA, Paranicas et al. (2005) investigated a single distant ENA population and concluded that a ∼*r* ^−2^ intensity decrease was the most important effect on the signal and that the CG effect was minor for the species, energies, and distances they were considering.

Here, it seems like the CG effect does not have a significant impact for the most part, as can be seen in Figure 6b. Neither of the two hydrogen energy bands shows an increase in intensity toward larger “flow angles” (i.e., when the corotating plasma moves toward the detector), the curves are rather flat with some variability at more extreme values where less data is available. We note that the flow angle values shown are limited to 50°–130° as the original data is limited to spacecraft elevations >50° above the equatorially corotating plasma. Oxygen observations do indeed show the expected dependency, with ENA intensities slightly increasing with flow angle; the effect is however very small and deemed negligible, especially for the qualitative analyses which these observations are mostly used for in the community. We hence conclude that at the spacecraft elevations >50° considered in this study the CG effect does not need to be taken into account for the determination of ENA intensities. At lower elevations, the effect will of course be stronger as is discussed in, for example, Paranicas et al. (2005), but equatorial projections should not be used from these perspectives anyway.

## 8 Conclusions

In this study, we presented a new data set containing equatorial projections of Cassini‐INCA's ENA observations of Saturn's magnetosphere which is made available online for use in further scientific studies. We described the processing algorithm applied to the original raw data, including calibration and projection procedures, and summarized the resulting data set. Different types of data contamination have been identified and contamination events have been labeled in the final data product, paving the way for easier analyses of these valuable observations. We further investigated the effects of the projection procedure applied to the original data and highlight different aspects which should be kept in mind when working with the projections we provide. This includes discussions on the low INCA count rates, the scaling of ENA intensity with line of sight distance, and the impact of the CG effect.

A first use case of this set is presented in Kinrade, Bader, et al. (2020), where we investigate the statistical distribution of ENAs in Saturn's equatorial plane. We hope that this processed data set will improve the accessibility of Cassini‐INCA ENA observations and stimulate further important research on the dynamics of Saturn's magnetosphere.

## Data Availability

All Cassini‐INCA data used in this study are available on NASA's Planetary Data System (PDS) (https://pds.jpl.nasa.gov/). INCA ENA projections generated in this study are accessible at https://doi.org/10.17635/lancaster/researchdata/384.
